# Hypoglycemia as a potential risk for patients taking clopidogrel: A systematic review and meta-analysis

**DOI:** 10.3389/fendo.2023.1091933

**Published:** 2023-02-28

**Authors:** Shi Chen, Jiaqi Qiang, Yuelun Zhang, Bin Zhao, Ran Tian, Tao Yuan, Ming Li, Mei Li, Yuxiu Li, Huijuan Zhu, Hui Pan

**Affiliations:** ^1^ Key Laboratory of Endocrinology of National Health Commission, Department of Endocrinology, Peking Union Medical College Hospital, Chinese Academy of Medical Sciences and Peking Union Medical College, Beijing, China; ^2^ Eight-Year Program of Clinical Medicine, Chinese Academy of Medical Sciences and Peking Union Medical College, Beijing, China; ^3^ Medical Research Centre, Peking Union Medical College Hospital, Chinese Academy of Medical Sciences and Peking Union Medical College, Beijing, China; ^4^ Department of Pharmacy, Peking Union Medical College Hospital, Chinese Academy of Medical Sciences and Peking Union Medical College, Beijing, China; ^5^ Department of Cardiology, Peking Union Medical College Hospital, Chinese Academy of Medical Sciences and Peking Union Medical College, Beijing, China; ^6^ State Key Laboratory of Complex Severe and Rare Diseases, Peking Union Medical College Hospital, Chinese Academy of Medical Sciences and Peking Union Medical College, Beijing, China

**Keywords:** clopidogrel, hypoglycemia, insulin autoimmune syndrome, meta-analysis, adverse event

## Abstract

**Background:**

Clopidogrel is a cornerstone antiplatelet drug used in cardiovascular, cerebrovascular, and peripheral artery diseases. The sulfhydryl group of clopidogrel metabolite could induce insulin autoimmune syndrome (IAS) with hypoglycemia as the major symptom. Discontinuing clopidogrel and substituting it with ticagrelor has been revealed as an effective treatment in previous studies. Since hypoglycemia serves as a risk factor for cardiovascular and cerebrovascular events, we aimed to determine the association between hypoglycemia/IAS and clopidogrel and to investigate whether clopidogrel is a modifiable and causal risk factor of hypoglycemia/IAS.

**Methods:**

MEDLINE, Embase, Cochrane databases, and clinical trial registries were searched for randomized controlled trials (RCTs) of clopidogrel from inception to 28 February 2022. RCTs comparing clopidogrel with placebo or other antiplatelet drugs were eligible if meeting the inclusion criteria: 1) clopidogrel was administrated 75 mg qd orally as a long-term antiplatelet prescription at least for months, and 2) hypoglycemia-inducible drugs were not used in the control arm. One investigator abstracted articles and performed a quality assessment. Uncertainties were resolved by discussions with two investigators independently. Odds ratio (OR) and risk difference (RD) were calculated and performed with subgroup analyses. The pre-specified protocol was registered in PROSPERO (CRD42022299622).

**Results:**

Six trials with 61,399 participants in total fulfilled the criteria and were included in the meta-analysis. Clopidogrel might not be associated with higher hypoglycemia odds (OR 0.95, 95% CI 0.65 to 1.40). However, Asian participants (p = 0.0437) seemed more likely to develop clopidogrel-associated hypoglycemia. Clopidogrel-associated hypoglycemia occurred at the highest rate of 0.03% (RD −0.00023, 95% CI −0.00077 to 0.00031), and this increased to 0.91% (RD 0.00210, 95% CI −0.00494 to 0.00914) in an aging population and to 0.18% (RD 0.00040, 95% CI −0.00096 to 0.00177) when Asian ratio of the population was elevated.

**Conclusions:**

We raise the concern that clopidogrel might be a modifiable and causal risk factor of hypoglycemia. The Asian population might be more vulnerable and need additional care.

**Systematic review registration:**

https://www.crd.york.ac.uk/prospero, identifier CRD42022299622.

## 1 Introduction

Hypoglycemia is a relatively uncommon condition with non-specific symptoms; however, its occurrence can be associated with daily performance disruption and physical injury involving the cardiovascular and central nervous systems (1, 2). Morbidities include myocardial ischemia, myocardial infarction, cardiac arrhythmia, transient ischemic attack, stroke, coma, seizure, cognitive impairment, and dementia (3, 4). Insulin autoimmune syndrome (IAS) is a cause of hypoglycemia characterized by hyperinsulinism and elevation in insulin autoimmune antibodies (IAAs), which might be triggered by drugs with sulfhydryl groups. The incidence is higher in populations with genetic background of susceptible human leukocyte antigen (HLA) alleles (1).

Clopidogrel is an anti-platelet drug prevalently prescribed for cardiovascular and cerebrovascular diseases, including acute coronary syndrome, myocardial infarction, stroke, and peripheral arterial disease. After activation in the hepatic P450 system, it transforms into a thiol derivative with a sulfhydryl group. As reported in previous cases (5), clopidogrel could induce IAS and trigger severe episodic hypoglycemia, making the drug threatening for the patients’ primary diseases. The phenotype frequency of HLA-DRB1*0403–one of the most IAS-susceptible alleles—is 6.94% in Asians and 2.07% in Caucasians (6, 7) (calculation method is described in the Materials and Methods section). Given that the cardiovascular disease is a global burden and among the top leading causes of death (8, 9), it is reasonable to postulate there are several millions of individuals carrying susceptible HLA alleles and taking clopidogrel at the same time. Among this possible affected population, clopidogrel plays the role of a double-edged sword.

In this article, we were the first to raise the concern that if clopidogrel induced IAS in such a population, the harm could exacerbate the state of illness and even threaten their life. However, in previous studies comparing clopidogrel with other antiplatelet drugs, whether hypoglycemia was associated with clopidogrel remained undetermined. Whether hypoglycemia was more prevalent in populations with a higher frequency of susceptible HLA genotypes, such as Asians, also lacked evidence. Therefore, it was urgent to find out if clopidogrel was a risk factor for hypoglycemia through a systematic review and meta-analysis of randomized controlled trials (RCTs) of clopidogrel.

## 2 Materials and methods

### 2.1 Search strategy

The meta-analysis was conducted according to a pre-specified protocol (PROSPERO: CRD42022299622). RCTs comparing clopidogrel to other antiplatelet drugs or placebos were searched in clinical registries and published literature. Terms (clopidogrel OR Plavix) were searched on ClinicalTrials.gov, www.controlled-trials.com, and www.clinicaltrialsregister.eu from inception to 16 February 2022. The syntax (clopidogrel OR Plavix) was searched in free-text and MeSH terms on PubMed, Embase, and the Cochrane Library from inception to 28 February 2022. The search strategy in PubMed was as follows: ((“clopidogrel”[Title/Abstract] AND “clopidogrel”[MeSH Terms]) OR “plavix”[Title/Abstract]) AND (meta-analysis[Filter] OR meta analysis[Filter] OR systematic review[Filter]). Meta-analyses and systematic reviews were filtered out (see details in **Supplementary Method**). No restriction was set on language or publication time. For each search result, those meeting these criteria were included: a) systematic review or meta-analysis on RCTs of clopidogrel and b) clopidogrel was compared to placebo or other antiplatelet drugs. Those meeting these criteria were excluded: a) duplicated publications; b) clopidogrel was used in both groups; c) meta-analyses or systematic reviews on pharmacokinetics, pharmacodynamics, pharmacogenetics, or cost-effective studies; and d) *in vitro* or animal studies. Then, for each eligible meta-analysis and systematic review, abstracts and full texts were retrieved to screen and extract RCTs included in the search process.

### 2.2 Selection criteria

For each search result in the clinical registries and RCT extracted from the literature, we reviewed results displayed on the websites and related publications. Trial inclusion criteria were as follows: a) since trials with small sample size may have selection bias and overestimate the effect, we included trials recruiting 100 or more participants in total; b) hypoglycemia-inducible antiplatelet (or anticoagulant) drugs were not used in the control arm; c) since the optimal administrating dose of clopidogrel remained undetermined with variable and non-standard in children, we included trials enrolling adults (age ≥18 years); d) clopidogrel was administrated 75 mg qd orally as a long-term antiplatelet prescription at least for months; e) patients’ combination drugs were balanced in the clopidogrel arm and the comparing arm; and f) the trial provided information on the occurrence of hypoglycemia. Drugs meeting these criteria were considered as hypoglycemia-inducible: a) chemical structures containing sulfhydryl groups and b) had been reported to induce hypoglycemia at therapeutic dose in literature (electronic searching strategy: generic name AND brand name AND (hypoglycemia OR hypoglycaemia OR (insulin autoimmune syndrome)).

### 2.3 Data extraction and quality assessment

A standard data extraction form was used to extract baseline characteristics, interventions, and adverse events amount of hypoglycemia. Other associated hypoglycemic symptoms, such as coma, seizure, and shock, were not counted. The quality of included trials was assessed using the Cochrane Collaboration risk-of-bias tool (10). Literature search, review, data extraction, and risk assessment were performed by JQ. Uncertainties were resolved by discussing with SC and consulting a third investigator (YZ) for arbitration.

### 2.4 Data synthesis and analysis

Publication bias was tested with a funnel plot and Egger’s test (11). To quantify the potential risk of hypoglycemia in clopidogrel compared to control groups, odds ratios (ORs) and associated 95% confidence intervals (CIs) were first calculated using the random-effects model and the Mantel–Haenszel method. Statistical heterogeneity among trials was evaluated with *I* ^2^ statistics (12). Subgroup analyses of OR were performed by comparing clopidogrel with drugs in the control arm individually (ticagrelor, edoxaban, aspirin + extended-released dipyridamole, and placebo). In meta-regression analyses, the association between covariates (mean age, female ratio, Caucasian ratio, Asian ratio, and follow-up duration) and the OR was investigated using the DerSimonian–Laird method to estimate the between-study variance Tau^2^ and the Hartung–Knapp method to adjust type I error. Sensitivity analyses were conducted by excluding trials with placebo, restricting to trials using aspirin as background treatment, using Peto’s method to calculate the random-effects estimate of OR, and not using the Hartung–Knapp method adjustment in meta-regression.

To estimate the numbers needed to harm, a meta-analysis was next performed on risk difference (RD) by using the random-effects model and Mantel–Haenszel method. Subgroup analyses were performed using the median as the division boundaries, including mean age (>66.6 and ≤66.6 years), female sex ratio (>28.6% and ≤28.6%), Caucasian ratio (>81% and ≤81%), and Asian race ratio (>12% and ≤12%). Individual comparisons were also performed on clopidogrel versus drugs in the control arm (ticagrelor, placebo, aspirin + extended-released dipyridamole, and edoxaban).

Statistical analysis was completed in R (version 4.0.0) with the “meta” (version 4.16.2) (13) package. Confidence intervals not containing values of 1.00 and two-sided p-values of less than 0.05 were considered statistically significant.

### 2.5 HLA frequency calculation

HLA phenotype frequency is the percentage of individuals carrying the specific HLA allele in the population. We searched allele frequency (AF) and phenotype frequency (PF) of susceptible HLA alleles carried by IAS patients in the Allele Frequency Net Database (AFND) (6, 7). Asian race incorporated data of “Oriental” and “Asian” of ethnic origin labels in selection choice; Caucasian race incorporated “Caucasoid” and “Hispanic”. For items with missing phenotype frequency, the formula PF = 1 − (1 − AF)^2^ was used.

## 3 Results

**Figure 1:**
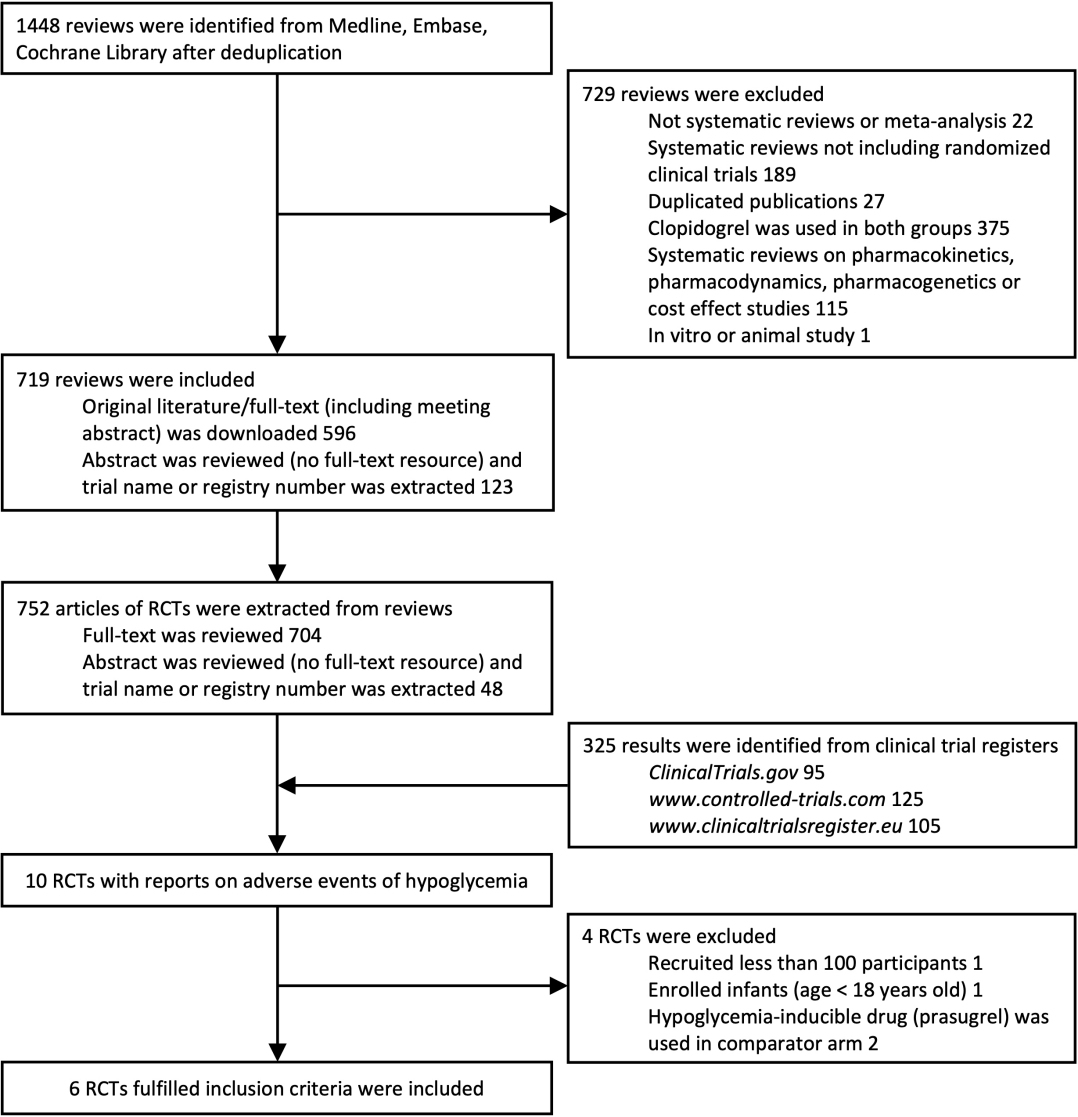
Flowchart of literature search to identify randomized controlled trials of clopidogrel.

A total of 752 publications of RCTs from databases and 325 RCTs from clinical trial registries were extracted. A total of 10 RCTs with reports of hypoglycemia were extracted. One trial that recruited less than 100 participants (NCT01864005), one trial that enrolled infants (NCT00396877), and two trials (14, 15) using prasugrel (containing sulfhydryl group in active metabolite) in the control arm were excluded. Six trials with 61,399 participants in total fulfilled the inclusion criteria and were included (**Figure 1**). Adverse events reported by 61,048 participants were included in the statistical analyses. Baseline characteristics are shown in **Table 1**. Risk-of-bias assessment identified some concerns for two of six trials (**Supplementary Table 1**). Egger’s test yielded p = 0.453, and a funnel plot was graphed (**Supplementary Figure 1**). All trials were distributed symmetrically around the vertical stripped line, showing no underlying publication bias.

**Table 1:** Baseline characteristics for participants in clopidogrel RCTs.

Trial	Publication year	Indication	Treatment	Follow-up period	Mean age (years)	Female sex (%)	Race (%)	
							Caucasian	Asian
EUCLID (16) NCT01732822 (N = 13,885)	2017	Peripheral artery disease	Ticagrelor 90 mg bid vs clopidogrel 75 mg od	30 months	66.6 (8.4)	28.0	0.81	0.12
ACTIVE A (17) NCT00249873 (N = 7,554)	2009	Atrial fibrillation vascular risk	Clopidogrel 75 mg od vs placebo od, all received aspirin 75 to 100 mg od	3.6 years	71.0 (10.2)	41.8	0.94	0.04
PLATO (18) NCT00391872 (N = 18,624)	2009	Acute coronary syndrome	Ticagrelor 90 mg bid vs clopidogrel 75 mg od, all received aspirin 75 to 100 mg od	13 months	62.2 (22.4)	28.4	0.92	0.06
PHILO (19) NCT01294462 (N = 801)	2015	Acute coronary syndrome, percutaneous coronary intervention	Ticagrelor 90 mg bid vs clopidogrel 75 mg od, all received aspirin 75 to 100 mg od	13 months	67 (11)	23.5	0.00	1.00
PRoFESS (20) NCT00153062 (N = 20,332)	2008	Stroke	Aggrenox (aspirin 25 mg plus extended-release dipyridamole 200 mg) bid vs clopidogrel 75 mg qd, and telmisartan 80 mg/placebo qd	2.5 years	66.1 (8.6)	36.0	0.62	0.33
ePAD (21) NCT01802775 (N = 203)	2018	Peripheral arterial disease	Edoxaban 60 mg qd vs clopidogrel 75 mg qd, all received aspirin 100 mg qd	6 months	67.4 (9.5)	28.6	0.94	0.00

N, the number of participants in actual enrollment; RCT, randomized controlled trial.

**Figure 2:**
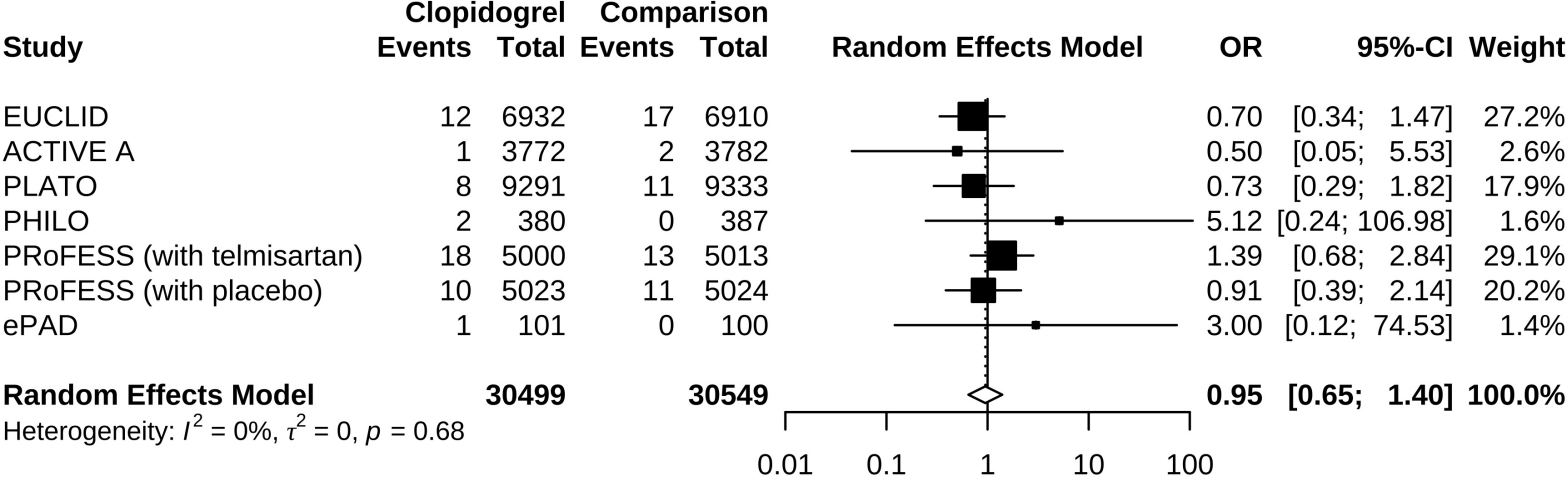
Forest plot of odds ratio estimation.

**Figure 3:**
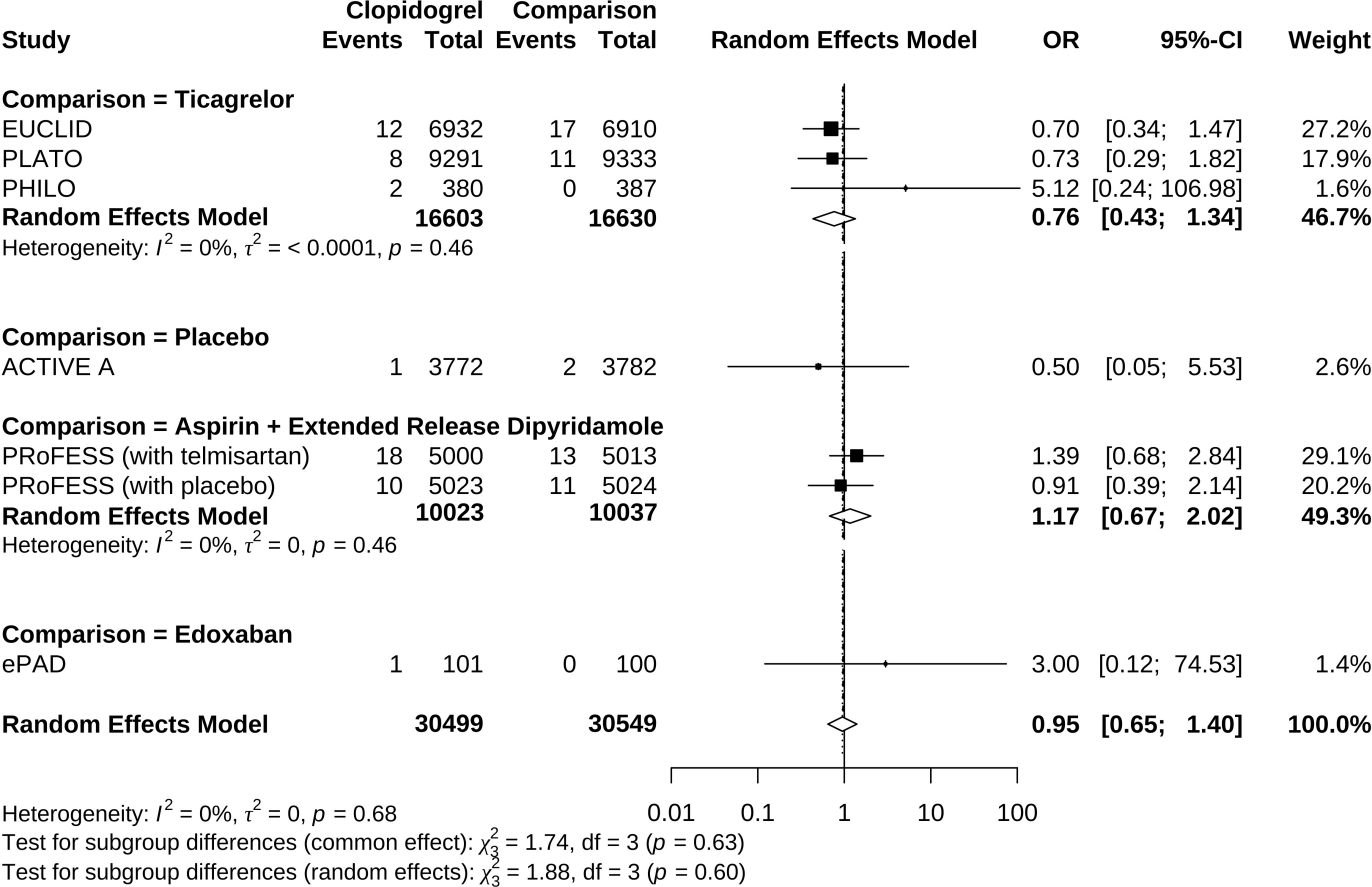
Forrest plot of odds ratio estimation in individual comparisons of clopidogrel versus drugs in the control arm.

**Figure 4:**
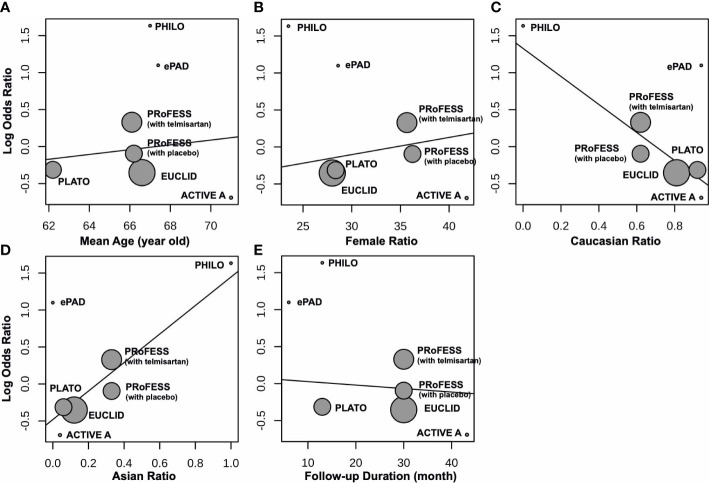
Meta-regression between clopidogrel-associated hypoglycemia and **(A)** mean age (years), **(B)** female ratio, **(C)** Caucasian ratio, **(D)** Asian ratio, and **(E)** follow-up duration (months).

Compared to control groups, clopidogrel was associated with 5% decreased odds (OR 0.95, 95% CI 0.65 to 1.40) of hypoglycemia (**Figure 2**). Test of heterogeneity yielded *I* ^2^ = 0%, indicating low heterogeneity between trials. We undertook mono-factor meta-regression analyses to identify potential sources of trial heterogeneity. In subgroup analyses comparing clopidogrel with drugs in the control arm, clopidogrel versus ticagrelor yielded OR = 0.76 (95% CI 0.43 to 1.34), clopidogrel versus edoxaban yielded OR = 3.00 (95% CI 0.12 to 74.53), clopidogrel versus aspirin + extended-released dipyridamole yielded OR = 1.17 (95% CI 0.67 to 2.02), and clopidogrel versus placebo yielded OR = 0.50 (95% CI 0.05 to 5.53) (**Figure 3**). Among the covariates, a higher Asian ratio was related to higher OR (p = 0.0437), whereas the others were not (age, p = 0.7486; female ratio, p = 0.5872; Caucasian ratio, p = 0.0501; follow-up duration, p = 0.8451) (**Figure 4**).

In sensitivity analyses, excluding trials with placebo (ACTIVE A) yielded OR 0.97 (95% CI 0.65 to 1.43); restricting to trials using aspirin as background treatment (ACTIVE A, PLATO, PHILO, and ePAD) yielded OR 0.87 (95% CI 0.39 to 1.93); using Peto method to calculate the random-effects estimate yielded OR 0.96 (95% CI 0.66 to 1.41); not using Hartung–Knapp method in meta-regression yielded no significant results of all covariates, with p = 0.7646 in age, p = 0.6157 in female ratio, p = 0.1315 in Caucasian ratio, p = 0.1246 in Asian ratio, and p = 0.8547 in follow-up duration.

**Figure 5:**
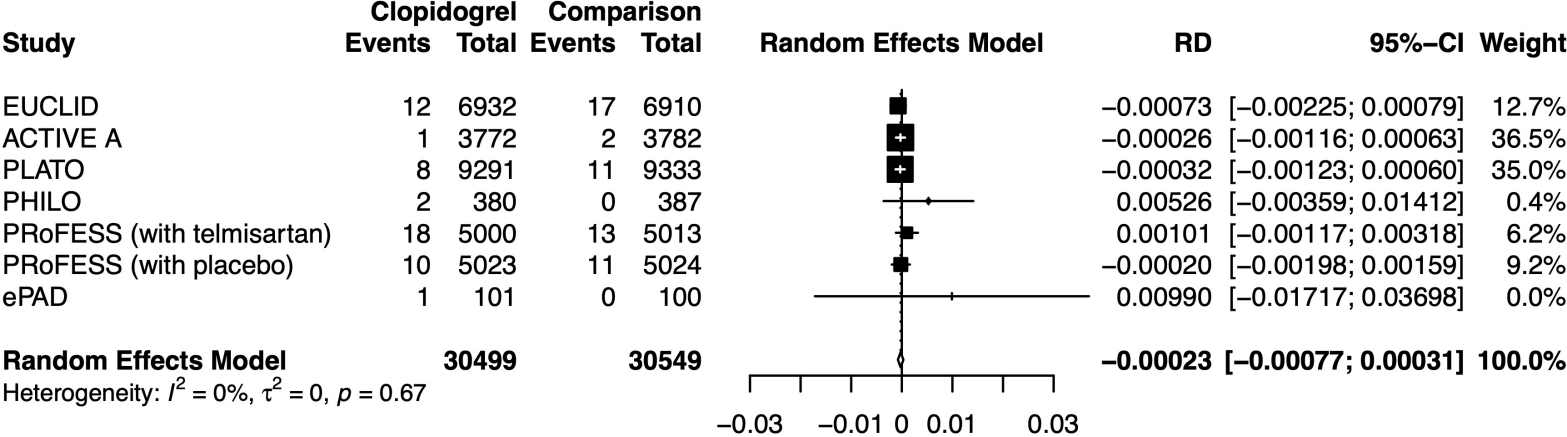
Forest plot of risk difference estimation.

**Figure 6:**
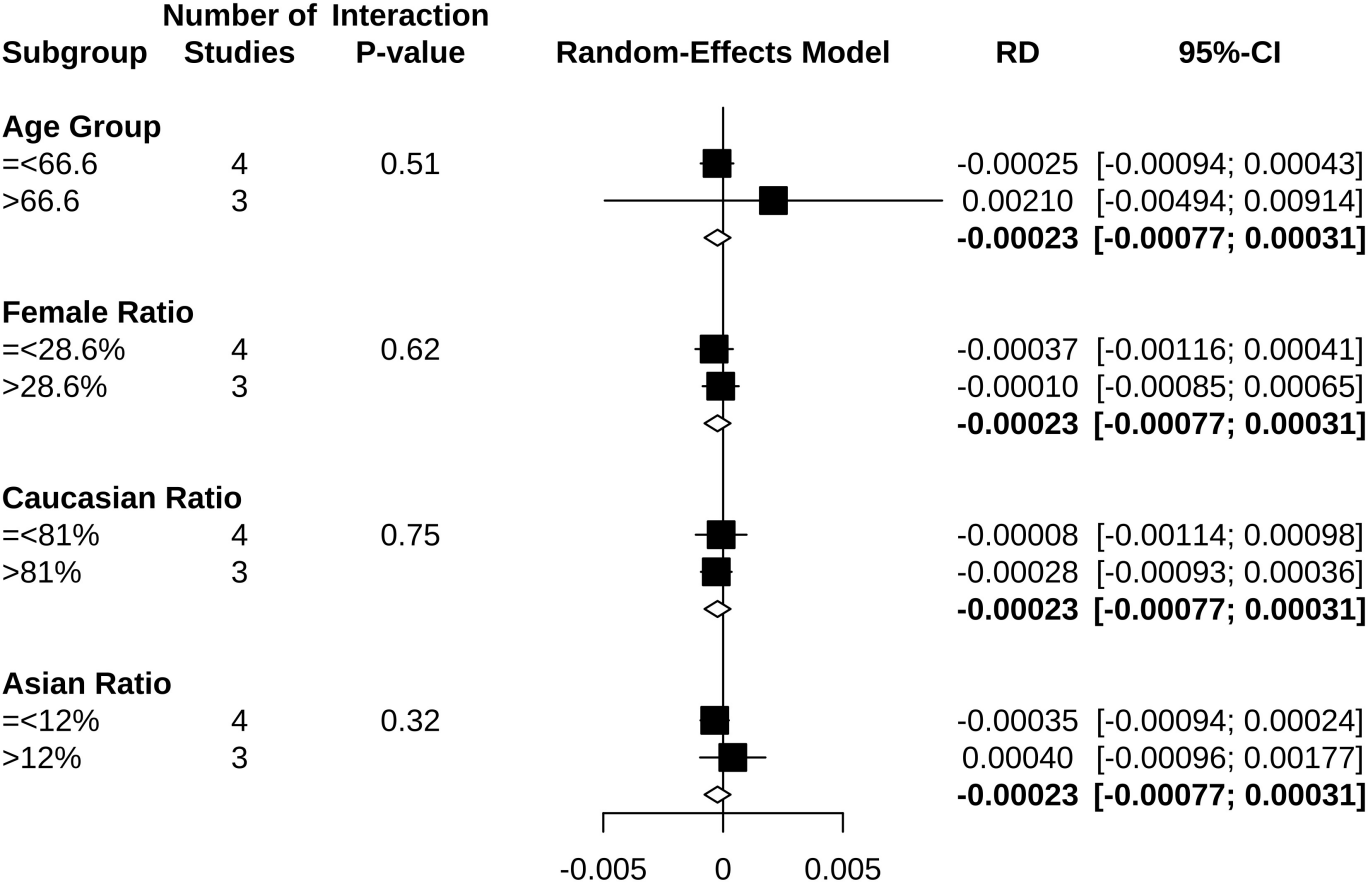
Forest plot of subgroup analysis in risk difference estimation.

**Figure 7:**
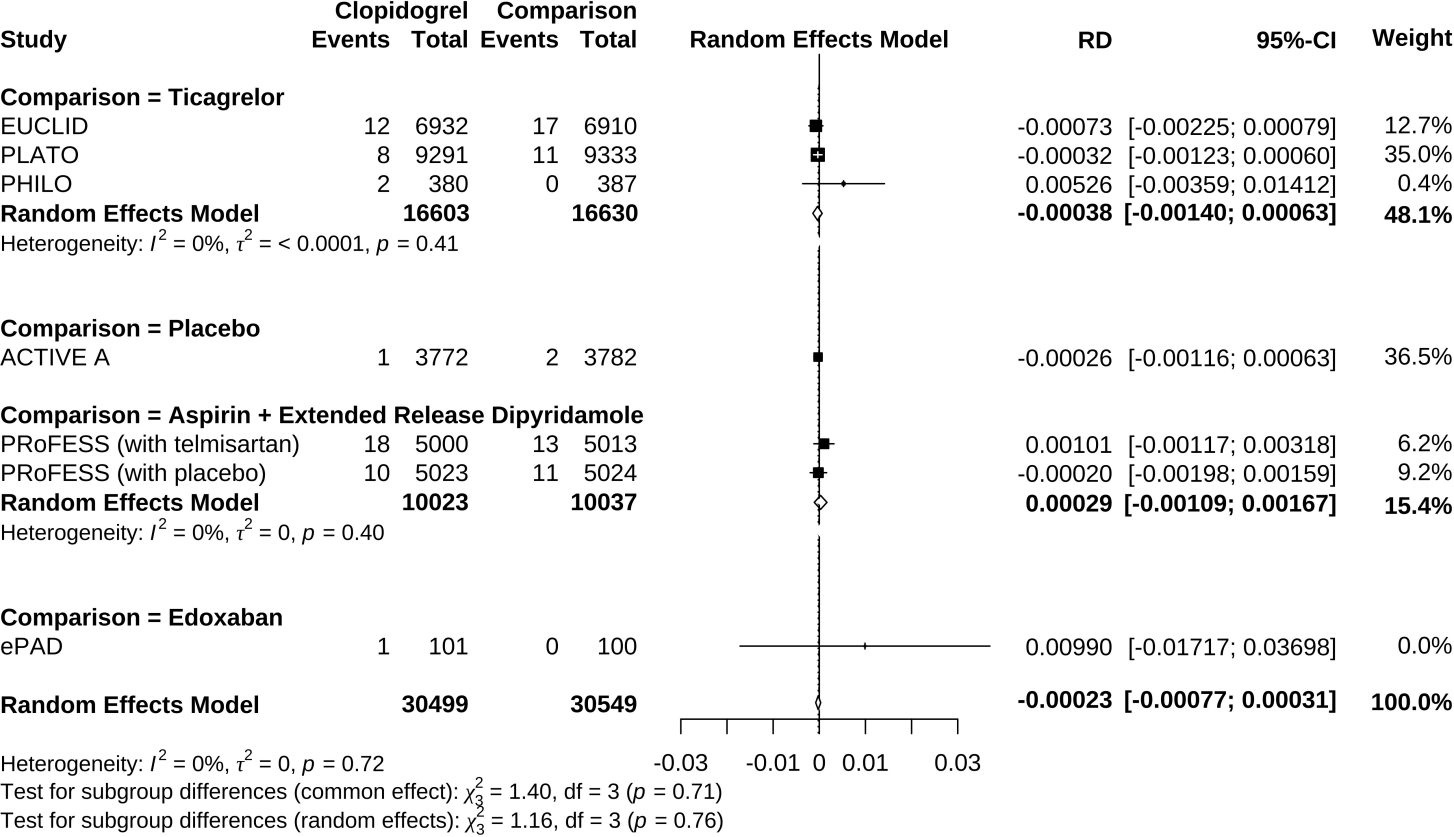
Forrest plot of risk difference estimation in individual comparisons of clopidogrel versus drugs in the control arm.

Clopidogrel decreased the risk of hypoglycemia incidence by 0.023% (RD −0.00023, 95% CI −0.00077 to 0.00031) (**Figure 5**). In a subgroup of age older than 66.6 years, clopidogrel increased the risk by 0.210% (RD 0.00210, 95% CI −0.00494 to 0.00914). In a subgroup with an Asian ratio higher than 12%, clopidogrel increased the risk by 0.040% (RD 0.00040, 95% CI −0.00096 to 0.00177), whereas the other subgroups did not present increasing RD (**Figure 6**). To interpret, one in at least 3,220 patients taking clopidogrel was under threat of hypoglycemia manifesting in IAS. If the average age of the population exceeded 66.6 years, the number was 109. If the Asian ratio of the population increased, the number was one in at least 565. In subgroup analyses comparing clopidogrel with drugs in the control arm, clopidogrel versus ticagrelor yielded RD = −0.00038 (95% CI −0.00140 to 0.00063); clopidogrel versus edoxaban yielded RD = 0.00990 (95% CI −0.01717 to 0.03698), clopidogrel versus aspirin + extended-released dipyridamole yielded RD = 0.00029 (95% CI −0.00109 to 0.00167), and clopidogrel versus placebo yielded RD = −0.00026 (95% CI −0.00116 to 0.00063) (**Figure 7**).

## 4 Discussion

This study focused on clopidogrel as a modifiable and causal risk of hypoglycemia manifesting in IAS. Since hypoglycemia deteriorates the risks of primary cardiovascular diseases, we conducted this research and generated the main findings, as follows: a) clopidogrel might not be associated with higher hypoglycemia risk, but Asians seemed more likely to develop clopidogrel-associated hypoglycemia; b) clopidogrel-associated hypoglycemia occurred at the highest rate of 0.03%, and this increased to 0.91% if it is an aging population and to 0.18% when Asian ratio of the population was elevated.

The mechanism for clopidogrel to trigger IAS has been revealed before. After ingestion, clopidogrel is transformed into a sulfhydryl derivative by hepatic enzymes CYP2C19, CYP2B6, CYP3A4, etc., which disrupts the disulfide bonds of the insulin molecule. The conformation-changed insulin molecule acquires immunogenicity and stimulates the proliferation of T cells and the production of IAAs. IAAs bind to insulin and dissociate in an unregulated way. When the insulin pool is discharged, blood glucose level decreases and induces hypoglycemia symptoms (22–24). In this autoimmune process, susceptible HLA alleles include HLA-DRB1*0406, DRB1*0403, and DQB1*0302 (25–27). In our calculation of HLA allele frequency (6), HLA-DRB1*0406 is carried by 3.59% Asians and 0.13% Caucasians, DRB1*0403 by 6.94% Asians and 2.07% Caucasians, and DQB1*0302 by 9.50% Asians and 30.40% Caucasians (calculation method is described in the Materials and Methods section).

Clopidogrel is commonly used in a massive number of cardiovascular, cerebral vascular, and peripheral artery disease patients. In the 2020 Medical Expenditure Panel Survey (MEPS) released by the Agency for Healthcare Research and Quality (AHRQ), the prescription of clopidogrel was 19,377,527, even higher than aspirin in the United States (28, 29). To multiply the prescription number and susceptible HLA allele frequency, the estimated number of victims is considerable. However, clopidogrel-induced IAS/hypoglycemia was rarely reported in either case or RCTs. Moreover, no systematic review or meta-analysis was conducted to reveal the association or evaluate the potential risks. The earliest case dated back to 2004, but the authors did not realize the triggering effect of clopidogrel on IAS (30). Until 2016, the concept of clopidogrel-induced IAS was formally proposed by Japanese scientists (22).

In the meta-analysis, clopidogrel appears to reduce the risk of hypoglycemia but with a non-confirmative trend. Result discrepancies between our postulation and meta-analysis could be attributed to several factors: a) hypoglycemia is a rare adverse event in traditional RCTs of non-diabetes mellitus participants and with strict recruitment criteria; b) clopidogrel metabolism depends on certain P450 enzyme, possibly not carried by all participants; c) participants of these trials were mainly Caucasians in their sixties, not balanced in age and race, whereas Asians are more susceptible to IAS due to genetic background; d) since hypoglycemia emerges asymptomatically or symptomatically with variable manifestation and onset time, it might not be identified during follow-up; e) some IAS are self-limiting and thus not reported (31).

Trials with higher Asian ratios seemed to have higher OR of clopidogrel-associated hypoglycemia, suggesting that Asians were probably more vulnerable. Consistently, geographic distribution was observed in IAS in previous studies (32). Asians, especially Japanese people, carrying HLA-DRB1*0406 at a higher frequency were more likely to develop IAS (33). This was validated by higher Asian carrier proportions of DRB1*0406 and DRB1*0403 alleles from our calculation (32). Although we also observed a considerable frequency of susceptible HLA allele carried by Caucasians, the odds and risks of clopidogrel-induced hypoglycemia remain evidence-less in this population based on the meta-analysis result. Additional care might not be given to these patients.

As the association of clopidogrel and IAS/hypoglycemia is supported by biological plausibility and temporal relationship, we believe it necessary to estimate patients to be affected. One in at least 3,220 people (0.031%) in all patients, one in at least 109 people (0.91%) in the population with an average age above 66.6 years, and one in at least 525 people (0.18%) in higher-Asian-ratio subgroups might be affected by clopidogrel-induced hypoglycemia. Considering the massive sales of clopidogrel, we estimated that the actual number under threat was huge. Also, relatively rare adverse events have been drawing attention due to their serious outcomes, for instance, rhabdomyolysis induced by statins (at a risk of one in 66,667) (34) and allopurinol hypersensitivity syndrome (AHS) induced by allopurinol (at a risk of one in 1,000) (35).

Since clopidogrel administration is a modifiable and causal risk factor, drug discontinuation is the first-line therapy when hypoglycemia manifests. Drug substitution after clopidogrel discontinuation is also essential to maintain antiplatelet control over cardiovascular disease risk in treatment. The situation also depends on the clinical indications. For patients with non-ST-segment elevation acute coronary syndrome, a previous pharmacovigilance study based on data mining of the Food and Drug Administration Adverse Event Reporting System (FAERS) proposed ticagrelor as an alternative to substitute clopidogrel (36). Individual comparisons of clopidogrel versus drugs in the comparing arm in this meta-analysis might also provide information on which drug to switch to. Clopidogrel has higher (but not statistically significant) odds and risks than edoxaban and aspirin + dipyridamole to induce hypoglycemia while lower odds and risks (but not statistically significant) than ticagrelor. These results suggested that edoxaban is a possible substitute for patients with peripheral artery disease undergoing endovascular treatment and that aspirin + dipyridamole is a possible substitute for patients with ischemic stroke. Since our result lacked statistical significance, the inconsistent effect of clopidogrel *vs.* ticagrelor in the previous study probably needed further validation.

Although we revealed the association between hypoglycemia and clopidogrel according to widely acknowledged standards and guidelines, there were some limitations to mention. First, only aggregate data were available in included RCTs, whereas individual participant data (IPD) provide a more precise estimation of baseline characteristic effects. However, previous methodological research demonstrated that IPD and traditional aggregate data meta-analysis do not differ much in the combined effects (37). Second, observational studies were not included in our meta-analysis, so some cases with clopidogrel-induced IAS reported in previous publications may be missed in our evidence synthesis. Third, missing data might come from RCTs of clopidogrel published but not included in any of our searched clinical registries, meta-analysis or systematic review; or might have been published without reporting hypoglycemia. Fourth, the study population might contain more participants of certain races from developed countries that carried out more RCTs. Since hypoglycemia of IAS has geographical and racial distribution differences, the over-representation of participant races might lead to inaccurate estimation. Last, all included trials reported hypoglycemia instead of IAS. There was the possibility that some hypoglycemia events were not IAS manifestations. Moreover, to avoid duplication, reports on hypoglycemia-associated symptoms were excluded.

In summary, we were the first to explore the potential risk of IAS/hypoglycemia triggered by clopidogrel and estimated the incidence rate. We also pointed out that the Asian population might need extra attention. Clopidogrel substitution with alternative antithrombotic drugs might be effective in treatment. Future studies are expected to provide more robust evidence on the association between clopidogrel and IAS/hypoglycemia. We also recommend that hypoglycemia and IAS are specified as secondary endpoints in future large endpoint clopidogrel trials. We believe this study will arouse physicians’ consciousness of hypoglycemia occurrence in prescribing clopidogrel and provide practical benefits for patients taking clopidogrel from a novel perspective.

## Data availability statement

The original contributions presented in the study are included in the article/**Supplementary Material**. Further inquiries can be directed to the corresponding author.

## Author contributions

SC, BZ, RT, TY, MiL, MeL, YL, and HZ conceived the study. SC, JQ, and YZ designed the study. JQ and YZ collected the systematic review data. SC, JQ, YZ, and HP analyzed and interpreted the data. SC, JQ, and YZ drafted the manuscript. All authors revised and approved the final version of the manuscript. All authors had full access to all the data in the study. HP accessed and verified the underlying data and had final responsibility for the decision to submit for publication. All authors contributed to the article and approved the submitted version.
