# Supplementary material for: Hypoglycemia as a potential risk for patients taking clopidogrel: A systematic review and meta-analysis
Source: Front Endocrinol (Lausanne). 2023 Feb 28;14:1091933. doi: 10.3389/fendo.2023.1091933 (PMC10011644; doi:10.3389/fendo.2023.1091933)
Supplement: Supplementary file 1 [file DataSheet_1.docx]

Supplementary Material

# Supplementary Method: electronic searching strategy

## Cochrane Library (up to February 28^th^ 2022)

#1 (clopidogrel):ti,ab,kw OR (plavix):ti,ab,kw

Filter: Cochrane Reviews 15

## PubMed (up to February 28^th^ 2022)

Search: ((clopidogrel[Title/Abstract])) AND (clopidogrel[MeSH Terms]) OR "plavix"[Title/Abstract])

Filters: Meta-Analysis, Systematic Review

Term: (("clopidogrel"[Title/Abstract] AND "clopidogrel"[MeSH Terms]) OR "plavix"[Title/Abstract]) AND (meta-analysis[Filter] OR meta analysis[Filter] OR systematic review[Filter]) 337

## Embase (up to February 28^th^ 2022)

#1 'plavix':ti,ab OR 'clopidogrel':ti,ab 25,710

#2 'meta analysis'/de OR 'meta analysis topic'/de OR 'systematic review'/de 465,726

#3 #1 AND #2 1383

# Supplementary Figures and Tables


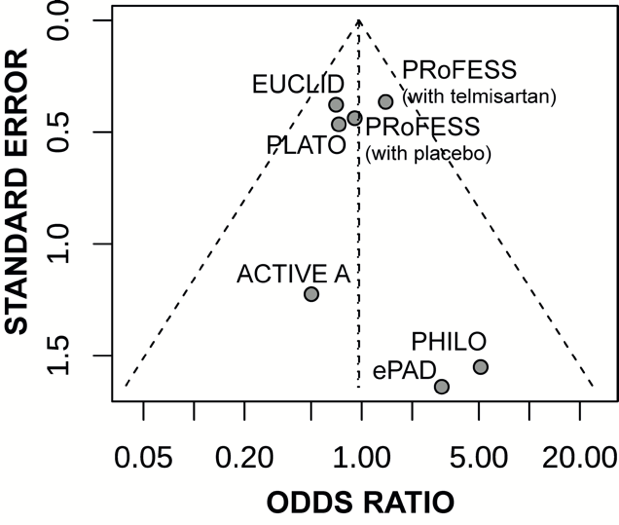


**Supplementary Figure 1.** **Funnel plot to demonstrate heterogeneity between trials.**

**Supplementary Table 1. Risk of bias of included trials assessed by the Cochrane risk of bias assessment tool.**

|  | **Sequence** **generation** | **Allocation** **concealment** | **Blinding** **of** **and** **personnel participants** | **Blinding** **of** **outcome** **assessment** | **Incomplete** **outcome data** | **Selective** **reporting** | **Other Bias (baseline balance)** |
| --- | --- | --- | --- | --- | --- | --- | --- |
| EUCLID | L | L | L | L | L | L | L |
| ACTIVE A | L | L | L | L | L | L | L |
| PLATO | L | L | L | L | L | L | L |
| PHILO | L | L | L | L | L | L | U |
| PRoFESS | L | L | L | L | L | L | L |
| ePAD | L | L | U | L | L | L | L |

L = low risk of bias; U = unclear risk of bias.
